# Mutations in *Bcl10* are very rare in colorectal cancer

**DOI:** 10.1038/sj.bjc.6690562

**Published:** 1999-07

**Authors:** J G Stone, A J Rowan, I P M Tomlinson, R S Houlston

**Affiliations:** Section of Cancer Genetics, Institute of Cancer Research, Sutton, Surrey SM2 5NG, UK; Molecular and Population Genetics Laboratory, Imperial Cancer Research Fund, 44, Lincoln's Inn Fields, London WC2A 3PX, UK

**Keywords:** colorectal cancer, *Bcl10*

## Abstract

*Bcl10* is a recently identified gene reported to be involved commonly in human malignancy (Willis et al (1999) *Cell* 96: 1–20). To investigate whether it is frequently mutated in colorectal cancer we have analysed a series of 132 colorectal cancers and eight colorectal cancer cell lines for mutations in *Bcl10*. One feature of the *Bcl10* gene is that it harbours two polyadenine tracts. These repeating elements in genes can be prone to a high rate of mutation if there is defective mismatch repair. To examine the possibility that *Bcl10* may be preferentially mutated in mismatch repair-deficient cancers, 49 of the tumours and cell lines were known to be replication error (RER)-positive and, of these, ten were from individuals harbouring germline mutations in *hMLH1* or *hMSH2*. No pathogenic mutations were detected in the tumours and only one mutation was found in the colorectal cancer cell lines. These results indicate that *Bcl10* is unlikely to be involved in the pathways of colorectal carcinogenesis. © 1999 Cancer Research Campaign


*British Journal of Cancer* (1999) **80**(10), 1569–1570
© 1999 Cancer Research Campaign
Article no. bjoc.1999.0562

### Mutations in *Bcl10* are very rare in colorectal cancer

**JG Stone[1], AJ Rowan[2], IPM Tomlinson[2] and RS Houlston[1]**

[1]Section of Cancer Genetics, Institute of Cancer Research, Sutton, Surrey SM2 5NG, UK; [2]Molecular and Population Genetics Laboratory, Imperial Cancer Research Fund, 44, Lincoln's Inn Fields, London WC2A 3PX, UK

**Summary** *Bcl10* is a recently identified gene reported to be involved commonly in human malignancy (Willis et al (1999) *Cell* 96: 1–20). To investigate whether it is frequently mutated in colorectal cancer we have analysed a series of 132 colorectal cancers and eight colorectal cancer cell lines for mutations in *Bcl10*. One feature of the *Bcl10* gene is that it harbours two polyadenine tracts. These repeating elements in genes can be prone to a high rate of mutation if there is defective mismatch repair. To examine the possibility that *Bcl10* may be preferentially mutated in mismatch repair-deficient cancers, 49 of the tumours and cell lines were known to be replication error (RER)-positive and, of these, ten were from individuals harbouring germline mutations in *hMLH1* or *hMSH2*. No pathogenic mutations were detected in the tumours and only one mutation was found in the colorectal cancer cell lines. These results indicate that *Bcl10* is unlikely to be involved in the pathways of colorectal carcinogenesis.

**Keywords:** colorectal cancer; *Bcl10*

In 1990, Fearon and Vogelstein proposed that a series of specific mutations occurs in the development of colorectal cancers. These mutations are associated with histological features of tumours. Several postulates of the model appear to be correct, particularly the step-wise accumulation of genetic changes and the involvement of mutations in *APC* and *TP53* (Ilyas and Tomlinson, 1996). Since this model was published, it is now recognized that mutations within other genes are also involved. Moreover, there is evidence suggesting that some colorectal cancers develop along different molecular pathways (Ilyas and Tomlinson, 1996).

*Bcl10* is a recently identified gene, which appears to be commonly involved in the pathogenesis of a wide range of malignancies, including two colorectal cancer cell lines (Willis et al, 1999). To investigate whether *Bcl10* is commonly involved in colorectal carcinogenesis we have analysed 132 colorectal adenocarcinomas and eight colorectal cancer cell lines for mutations.

### MATERIALS AND METHODS

### Tumour specimens

Paired blood and tumour specimens were collected from 132 patients with histologically proven colorectal adenocarcinomas (76 paraffin-embedded and 56 fresh-frozen samples) and eight colorectal cancer cell lines. Tumour samples were obtained with informed consent and ethical review board approval. The following clinical data were also available from patients: patient sex, age at presentation, tumour site and replication error (RER) status.

*Received 16 April 1999*
*Accepted 23 April 1999*

*Correspondence to:* RS Houlston

### DNA extraction

DNA was salt extracted from EDTA blood samples using a standard sucrose lysis method. Cell line and fresh-frozen tumour DNAs were extracted using the Nucleon kit (Anachem). Formalin-fixed and paraffin-embedded sections of colorectal cancers, prepared for routine histopathology, were used for DNA preparation. Three times 15-μm sections were cut onto double-sided, clear adhesive tape and placed on glass slides. These were lightly stained with toluidine blue and regions containing at least 60% tumour microdissected. Tumours were digested in buffer, 10 mM Tris–HCl (pH 7.5), 1 mM EDTA, 15% (w/v) sodium dodecyl sulphate (SDS) and 500 μg $ml^{-1}$ proteinase K, for 16 h at 56°C. DNA was then precipitated with sodium acetate and ethanol following phenol–chloroform extraction.

### Mutation screening

The search for mutations in each of the three exons of *Bcl10* was performed using conformation-sensitive gel electrophoresis (CSGE). For the paraffin-embedded carcinomas, the full coding sequence of *Bcl10* was amplified by five different PCR reactions using published primer sequences (Willis et al, 1999). CSGE was performed as reported by Gangulay et al (1993). All samples with band shifts were sequenced in duplicate and in forward and reverse orientations after re-amplification of the appropriate exon from genomic DNA in the polymerase chain reaction (PCR). Purified PCR products were sequenced using the ABI Ready Reaction Dye Terminator Cycle Sequencing Kit and the ABI377 Prism Sequencer. For the cell line and fresh-frozen tumour samples, exons 2A, 2B and 3A were amplified, so as to include both the region of the genes in which almost all somatic *Bcl10* mutations had been reported and the polyadenine tracts in the gene. These samples were screened by single-strand conformation polymorphism (SSCP) and by direct genotyping using the ABI377 sequencer to search for insertions and deletions.

### RESULTS AND DISCUSSION

One interesting feature of the *Bcl10* gene is that it harbours two polyadenine tracts (Willis et al, 1999). One of the two *Bcl10* mutations in colorectal cancer lines reported by Willis et al (1999) was an insertion of A within one of these polyadenine tracts. These repeating elements in genes can be prone to a high rate of mutation if there is defective mismatch repair, and this is observed in RER-positive tumours at loci such as transforming growth factor (TGF)-β type II receptor and *Bax* (Parsons et al, 1995). To examine the possibility that *Bcl10* may be preferentially mutated in mismatch repair deficient cancers, 49 of the tumours and cell lines analysed were known to be RER-positive and of these ten were from individuals harbouring germline mutations in *hMLH1* or *hMSH2*.

In the none of the 132 tumour samples was any pathogenic mutation identified, although two polymorphic variants were detected. One of these was a synonymous G to C substitution within codon 8 encoding leucine, present in 47/76 of the cancers (62%) analysed at this site. The other polymorphism, detected in 25/76 cancers (39%), was a G to C base substitution within intron 1 of the gene at nucleotide 58. There was no difference in the frequency of either of these variants between RER-positive and RER-negative colorectal cancers. One previously reported frameshift mutation (Willis et al, 1999) was found in the RER+ cell line LOVO at one of the polyadenine tracts, as a heterozygote with the wild-type allele, LOVO harbours a mutation in *hMSH2* and shows an exceptionally high level of instability at polynucleotide repeats. In the light of the results from other tumours and cell lines, there must be severe doubts that the *Bcl10* mutation in LOVO has any functional significance.

We cannot entirely exclude the possibility that a minority of *Bcl10* mutations have been missed, or cannot be detected by a PCR-based approach. However, under test conditions we have found that CSGE can detect all small insertions and deletions and 90% of single-base substitutions, and that SSCP can detect about 80% of all small-scale mutations. Confirmation of the efficiency of CSGE and SSCP techniques was provided by the fact that we were able to demonstrate a number of single-base substitution polymorphisms within the gene and the previously described mutation in LOVO. Therefore, it is improbable that we have failed to detect any coding mutations. This study provides strong evidence that *Bcl10* is not involved in colorectal carcinogenesis.

### ACKNOWLEDGEMENTS

We thank the patients that took part in this study and the Cancer Research Campaign for their support. Some of the work was conducted in the Jean Rook Sequencing Laboratory, which is supported by BREAKTHROUGH Breast Cancer, UK charity 328323.

### REFERENCES

Fearon ER and Vogelstein B (1990) A genetic model for colorectal tumourigenesis. *Cell* **61**: 759–767

Ganguly A, Rock MJ and Prockop DJ (1993) Conformation-sensitive gel electrophoresis for rapid detection of single-base differences in double-stranded PCR products and DNA fragments: evidence for solvent-induced bends in DNA heteroduplexes. *Proc Natl Acad Sci USA* **90**: 10325–10329

Ilyas M and Tomlinson IPM (1996) Genetic pathways in colorectal cancer. *Histopathology* **28**: 389–399

Parsons R, Myeroff LL, Liu B, Willson JK, Markowitz SD, Kinzler KW and Vogelstein B (1995) Microsatellite instability and mutations of the transforming growth factor beta type II receptor gene in colorectal cancer. *Cancer Res* **55**: 5548–5550

Willis TG, Jadayel DA, Du MQ, Peng H, Perry AR, Abdul-Rauf M, Price H, Karren L, Majekodumi O, Wlodarska I, Pan L, Crook T, Hamoudi R, Isaacson PG and Dyer MJS (1999) Bcl10 is involved in t(1;14)(p22;q32) of MALT B cell lymphoma and mutated in multiple tumor types. *Cell* **96**: 1–20

 *© 1999 Cancer Research Campaign*
